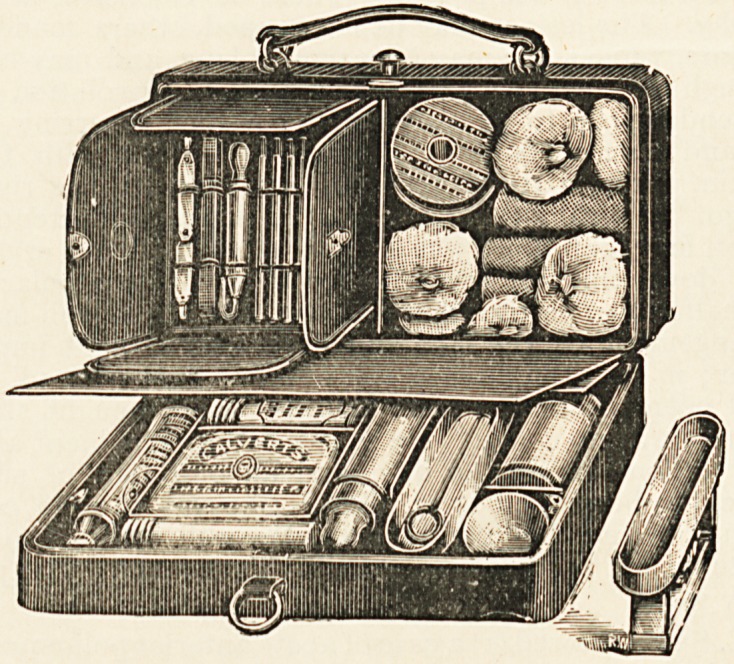# Notes on Preparations for the Sick

**Published:** 1899-06

**Authors:** 


### Notes on Preparations for the Sick.

**Diastol.**—THE STANDARD MALT EXTRACT CO. LIMITED, London.—The manufacture of this digestive is carried on at Clayton-le-Moors, Lancashire, by a special process, which gives excellent results. The diastasic power is high, and its use in many forms of dyspepsia gives good results, enabling cases of phthisis and other diseases of mal-nutrition to assimilate farinaceous foods in greater quantity than without it. In many cases of chronic dyspepsia we have found this preparation to be of real service. "If the starchy elements of the food were eliminated from the diet in a case of amyldyspepsia the alimentary troubles would soon cease," and so would the normal nutrition of the tissues unless the patient cared to run the greater risk of uric acid and urate poisoning. We think that the digestion, *even if artificial*, of the starchy foods is better than their elimination from the dietary.

**Varalettes: Citrate of Lithia; Citrate of Piperazine; Glycerophosphate of Lime; Urotropine; Potass. Citrate, Lithia Citrate; Soda Salicylate, Antipyrine, Caffeine pure; Vichy Salts.**—ALFRED BISHOP & SONS LTD., London.—These are the newer forms of Bishop's well-known preparations. The powder is compressed into what are called varalettes, which are said to have all the advantages of the tabloid combined with a greater solubility.

**Meta-Cresol-Anytol.**—GUSTAV HERMANNI, Jr., London.—This is specially prepared for the local treatment of diphtheria. It may be used as a three per cent. aqueous solution, either by inhalation or spray: the maker claims that it is non-irritating, is fatal to the cocci, and has a neutralising alterant action on the diphtheria toxin.

**Public Vaccinator's Bag.**—FERRIS & Co., Bristol.—In country districts it would be found convenient to carry such an assortment as is contained in this very complete and portable bag.

For use in towns the pocket case included in the bag will generally be found sufficient. To those who over a long number of years have vaccinated thousands of children with excellent results these cumbrous paraphernalia seem quite unnecessary. Their introduction very much resembles the employment of a steam-hammer for cracking a walnut. The people who are charged with the administration of vaccination laws and practice are very nearly succeeding in making the whole thing ridiculous. In the *British Medical Journal* for February 25th there is an excellent illustrated satire on the new methods.

## Figures and Tables

**Figure f1:**